# Quality of Life and Stigma in Epilepsy, Perspectives from Selected Regions of Asia and Sub-Saharan Africa

**DOI:** 10.3390/brainsci8040059

**Published:** 2018-04-01

**Authors:** Warren Boling, Margaret Means, Anita Fletcher

**Affiliations:** 1 Department of Neurosurgery, Loma Linda University, Loma Linda, CA 92354, USA; 2 School of Medicine, University of Louisville, Louisville, KY 40202, USA; margaret.means@louisville.edu; 3 Department of Neurology, University of Louisville, Louisville, KY 40202, USA; anita.fletcher@louisville.edu

**Keywords:** medically intractable epilepsy, surgery of epilepsy, stigma, quality of life, developing world

## Abstract

Epilepsy is an important and common worldwide public health problem that affects people of all ages. A significant number of individuals with epilepsy will be intractable to medication. These individuals experience an elevated mortality rate and negative psychosocial consequences of recurrent seizures. Surgery of epilepsy is highly effective to stop seizures in well-selected individuals, and seizure freedom is the most desirable result of epilepsy treatment due to the positive improvements in psychosocial function and the elimination of excess mortality associated with intractable epilepsy. Globally, there is inadequate data to fully assess epilepsy-related quality of life and stigma, although the preponderance of information we have points to a significant negative impact on people with epilepsy (PWE) and families of PWE. This review of the psychosocial impact of epilepsy focuses on regions of Asia and Sub-Saharan Africa that have been analyzed with population study approaches to determine the prevalence of epilepsy, treatment gaps, as well as factors impacting psychosocial function of PWE and their families. This review additionally identifies models of care for medically intractable epilepsy that have potential to significantly improve psychosocial function.

## 1. Literature Review

### 1.1. Prevalence of Epilepsy

Epilepsy is an important public health problem representing 0.6% of the global burden of disease [1] that particularly impacts the people living in the lowest income countries where epilepsy incidence may be 10 fold more than in the developed world. The prevalence of epilepsy is about 0.8% in North America [2]. However, prevalence rates have been estimated to vary from 49 to 215 per 100,000 people among regions of Africa [3], and an overall prevalence of 15 in 1000 has been described for Sub-Saharan Africa (SSA) [4]. In Asia, median prevalence has been observed to be similar to that found in Europe and North America, although with wide variation and with significant prevalence differences between rural and urban dwellers [4]. 

Sub-Saharan Africa includes 11% of the world population, but benefits from only 1.3% of world income, and of the least wealthy countries in the world, 80% are in Africa [5]. With this wealth disparity as a backdrop, it is understandable that many Sub-Saharan residents experience inadequate access to epilepsy care. Additionally, Sub-Saharan Africa is a mostly rural dwelling population, and of the about 36% of Africans who live in cities, most live in extreme poverty situations. A resource poor environment combined with inadequate access to medical care may explain the very high epilepsy prevalence rates observed in Africa, confounding these challenges this difficult environment results in significant difficulty determining the true epidemiology of the disease [5]. However, several authors have undertaken population studies of epilepsy in isolated regions of Sub-Saharan Africa. In a cross-sectional study using a survey questionnaire of rural and urban dwelling population in Kampala, Uganda and surrounding countryside, Kaddumukasa, et al. identified an overall epilepsy prevalence of 13.3% [6]. Ae-Ngibise, et al. [7] conducted a cross-sectional survey of individuals residing in the middle part of Ghana. The authors determined that 10.1 per 1000 individuals residing in this West African region had active convulsive epilepsy. In rural Zambia a door-to-door survey was conducted by Birbeck, et al. to determine active epilepsy prevalence identified an adjusted prevalence of 12.5/1000 [8]. Age-specific prevalence rates were found to be highest among children 5–15 years old with a second smaller peak in the over 65-years old age group. Ngugi, et al. [9] undertook a population-based cross-sectional and case-control study of prevalence and risk factors of convulsive epilepsy in five African Health and Demographic Surveillance System centres (Kilifi, Kenya; Agincourt, South Africa; Iganga-Mayuge, Uganda; Ifakara, Tanzania; and Kintampo, Ghana) to evaluate the reasons for significant variation in estimates of prevalence in different African regions. Adjusted prevalence rates for convulsive epilepsy ranged from a high of 14.8 per 1000 in Tanzania to 7.0 per 1000 in South Africa. Several epilepsy-related risk factors were found to be associated with epilepsy prevalence. However, the epilepsy risk factor of most importance was exposure to parasites, and this risk factor likely explained most of the regional variation in epilepsy prevalence. 

Regional variation of prevalence rates has been identified in Asia as well. In Southern provinces of China, a door-to-door survey identified the prevalence of epilepsy active within the last year and the last five years of 2.8% and 3.7%, respectively, and the prevalence of epilepsy was significantly higher in rural areas [10]. Wang, et al. also identified a contrast in epilepsy prevalence between rural and urban provinces with higher epilepsy rates in more rural provinces of Heilongjiang and Ningxia and relatively lower epilepsy rates in the more urban province of Henan [11]. Epidemiology studies of epilepsy in Pakistan and India have identified twice the epilepsy prevalence in rural compared with urban areas (6.23 versus 3.04 per 1000), and this difference was not related to neurocysticercosis infection rates [12,13]. 

The full epidemiological burden of epilepsy is likely underestimated in the door-to-door survey methodology most commonly employed in population studies of epilepsy in the developing world. In this method of case ascertainment in population-based studies of epilepsy, a questionnaire is administered to screen for members of a household with epilepsy. However, the commonly used screening questionnaire [14] is most sensitive to detect generalized tonic-clonic seizures so that individuals with partial complex, dyscognitive, or myoclonic seizures who do not experience generalized convulsions will be under-recognized. Furthermore, epilepsy associated stigma may lead to a reluctance to admit to an epileptic disorder in a household resulting in further underestimation of the number of prevalent cases.

A common finding worldwide is higher epilepsy rates in rural dwelling populations. The causes are most likely multifactorial and related to the recognized factors that result in a treatment gap in the developing world such as lower socioeconomic status of rural dwelling people and less access to medical clinics, health care providers, and medications. Additional possible factors impacting higher rates of epilepsy in rural areas include exposure to environmental etiologies of epilepsy such as parasites and less knowledge in rural areas of medical treatment options and greater reliance on traditional healers.

### 1.2. Epilepsy Related Mortality and Morbidity

In addition to an elevated prevalence, mortality of intractable epilepsy is high worldwide and particularly so in Sub-Saharan Africa. Epilepsy in East Africa has a substantially elevated standardized mortality ratio of 7.2 times age-matched controls, among the highest of the poorest areas of the world [15]. Low socioeconomic status is associated with an elevated risk of death from epilepsy [16,17], and epilepsy more frequently causes death in Sub-Saharan Africa than in developed countries [6]. The causes of epilepsy-related death include the underlying epilepsy etiologies, medical complications of epilepsy, trauma, suicide, and sudden unexplained death in epilepsy [18,19,20]. In Africa, burns are commonly encountered resulting from ubiquitous open-hearth fires in the home, and burn injuries are disproportionately encountered in people with epilepsy [21,22]. Birbeck reported burns or falls requiring hospitalization in 31% of epilepsy patients in rural Zambia [23]. In South Africa, 11% of accidental scalds presenting to one burn unit were epilepsy related [24]. In Tanzania, a long term follow-up study found that over 50% of deaths in epilepsy patients were related to status epilepticus, burns or drowning [25]. 

### 1.3. Epilepsy Treatment Gap

The treatment gap in epilepsy can be defined as the proportion of people with epilepsy who require treatment but do not receive it, and this parameter has been proposed as a useful concept to compare quality of care of epilepsy patients across regions and countries [26]. Treatment gap is strongly influenced by socioeconomics of a region or country. Meyer, et al. identified for every one-level decrease in World Bank income category, the treatment gap increased by a factor of 1.55 [26]. In the developing countries of Sub-Saharan Africa and Latin America, up to 90% of people with epilepsy have been reported to receive inadequate treatment or no treatment at all [12]. 

In Asian countries, the treatment gap is broadly estimated to be 29–98%, with a gap for most countries between 50% and 80% [12]. In Yueyang County, Hunan province, China, a more rural region of China, a door-to-door epidemiological survey of epilepsy was conducted by Pi, et al. [27] who found the lifetime prevalence rate of epilepsy was 4.5%. In this survey area, 35.0% of the people with epilepsy (PWE) had never been diagnosed or treated, 57.3% of PWE received non-standard treatments, and only 7.7% of patients received standard treatment. The non-standard treatments included the use of Chinese medicine in 42.7%. The authors identified in this rural Chinese region that 93.4% of patients with active epilepsy in the last year had a treatment gap as high as 96.6%. Hu, et al. [28] conducted questionnaire-based interviews in rural Western China to identify individuals with active convulsive epilepsy. The estimated prevalence of convulsive epilepsy was found to be 1.8 per 1000 in this population with a treatment gap estimated to be 66.3%. The authors were able to identify that a majority of individuals with epilepsy had consulted a doctor but failed to receive or adhere to an appropriate treatment program. 

In Sub-Saharan Africa, the epilepsy treatment gap has not been adequately studied although the information we have points to the presence of a significant treatment gap. Koba Bora, et al. [29] identified in a charity run neuropsychiatric clinic in Lubumbashi, the second largest city in the Democratic Republic of Congo, the epilepsy treatment gap was above 67%. When asked to describe the cause of their epilepsy, 55.3% of patients or their families considered epilepsy to be of spiritual or religious origin. In a random cluster sampling survey, questionnaires were administered to individuals living in rural and urban parts of Rwanda to evaluate aspects of epilepsy in the country [30]. The investigators found a prevalence of 41/1000 of people having active epilepsy, and a treatment gap of 67.8% that consisted of 43% receiving no treatment and the remainder of the gap due to traditional healer treatment or a mixture of traditional and medical treatment. Mbuba, et al. conducted a cross-sectional survey and risk-factor analysis of the epilepsy treatment gap in Kilifi, Kenya [31]. PWE were identified in a cross-sectional survey to establish the prevalence of active convulsive epilepsy and to determine the presence of any treatment gap. In those with epilepsy, the investigators evaluated blood levels of antiepileptic drugs (AEDs) to corroborate the questionnaire results concerning medical treatment. The epilepsy treatment gap based on AEDs detected in blood samples was 62.4% and of the PWE who sought medical treatment, 77% were prescribed antiepileptic medications. The authors found that non-adherence to medical treatment was associated with negative beliefs and attitudes about epilepsy. South Africa is a unique country in Sub-Saharan Africa due to its economic size and high sophistication level of its institutions. The gross domestic product of South Africa is the third highest in Africa after Nigeria and Egypt. South Africa is generally considered to be the most advanced medical system in Africa regarding patient care delivery and training of healthcare professionals. Ngugi, et al. identified prevalence of active convulsant epilepsy in Sub-Saharan Africa to be lowest in Agincourt, South Africa and highest in Ifakara, Tanzania with a significant difference between the two [9].

Worldwide, particularly in low resource areas, a treatment gap is found commonly to be much higher in rural than in urban areas. Common themes are identified that contribute to a gap in treatment that includes poverty, a lack of access to AEDs, inadequate access to physicians trained to manage epilepsy, poor knowledge about epilepsy among the community and healthcare providers, failed models of healthcare delivery, as well as stigma of epilepsy arising largely from common misconceptions about epilepsy that it is contagious or has supernatural origins [23,32].

### 1.4. Stigma and Psychosocial Impact of Epilepsy

The burden of epilepsy in the developing world affects the individual with epilepsy, their family, and society in general. PWE are impacted by the physical dangers of a seizure that brings risk of injury and death. There is a psychosocial impact as well on PWE who may be stigmatized and marginalized in society due to epilepsy. A child with epilepsy in a low-resource family frequently cannot participate in the activities of family life such as collecting water for fear of drowning or cooking for fear of falling in the fire. Financial security is at risk due to the cost of epilepsy medication and treatment [33]. Additionally, some parents must leave their jobs and experience lost income in order to provide care for the epileptic child. Psychosocial development is affected by the stigma of epilepsy, which is present worldwide to some degree but is most visible in the developing world. In East Africa, epilepsy is frequently thought to result from demonic possession and a seizure is believed to be contagious. In Asia, misconceptions that epilepsy is hereditary is a common belief about the cause of epilepsy, which results in an inability to marry for the PWE and negatively impacts the entire family of PWE [34]. In both China and Vietnam, the lack of marriageability of PWE centers on two main issues: (1) the possibility that epilepsy is inheritable and so could be passed on to offspring, and (2) the perceived inability of PWE to carry out everyday living tasks and contribute adequately to the family economy [35]. The psychological burden of epilepsy in China was assessed by Wang, et al. [36] by asking PWE the question “What do you worry about most”. Eighty percent of responders replied: ‘‘when the next seizure might occur” and the second most frequent response was: ‘‘facing discrimination”. In rural areas, traditional beliefs shape the definitions and treatment of epilepsy, which results in patients and families seeking less Western medical treatment. In rural China, Wang et al. [11] found the attitudes towards PWE are mostly negative. About half of the population believed that PWE should not be employed, and epilepsy was often identified as a mental disorder equivalent to insanity. Even among highly educated individuals and community leaders in China, about half of people perceived epilepsy as a ‘terrible’ condition, and that the disease was not curable and may be hereditary [37]. Studies in China have identified high rates of stigma felt by PWE and their families [38]. In a survey of PWE in China, about 1/3 of Chinese with epilepsy thought they were treated differently by others because of their epilepsy and 50% chose to keep their epilepsy secret [39]. In both China and Vietnam, surveys of attitudes about PWE found most people thought PWE have low intelligence, negative changes in character, and are often unfit for school [35].

In East and South-central Africa, children with epilepsy often are prevented from attending school due to stigma. The epileptic child may be shunned from family meals. Stigma creates an environment that leads to few or no childhood friends or play [40]. Witchcraft and sorcery may be invoked to combat the suspected demonic origin of epilepsy, which has additional risks of injury for the PWE and additional costs from traditional healers often sought out and exhausted prior to medical solutions [41,42]. As a result, PWE frequently experience rejection and isolation due to commonly held misconceptions of epilepsy etiologies and transmissibility [43,44]. 

Many facets of life are impacted by epilepsy. Personal health security is threatened by epilepsy. Fear of injury, concern about the social consequences of having a seizure, and the stigmatization related to a diagnosis of epilepsy leads to social isolation. Baskind and Birbeck have described three forms of stigmatization associated with epilepsy: enacted, felt, and courtesy [45]. Enacted stigma occurs when the source of discrimination is another person. Felt stigma results from a fear of being discriminated against. Courtesy stigma occurs when someone close to a PWE, in relation or proximity, feels stigmatized. Studies have indicated that nearly half, and sometimes as many as 70%, of PWE report feeling stigma [46,47,48,49]. PWE who report greater felt stigma are more likely to suffer from low self-esteem, poorer psychological function, and more uncertainty about the future [50]. Psychiatric comorbidities, including depression and anxiety, are more prevalent in PWE who report greater felt stigma [51,52,53,54]. Importantly, quality of life is appreciably decreased in these individuals [53,54]. In fact, psychosocial factors related to the stigma of epilepsy have a greater impact on the quality of life of PWE than clinical variables, such as side effects of medications [54,55]. The elements contributing to felt stigma vary by region and culture, but those commonly cited include seizure worry, lack of social support, and seizure severity [56,57,58,59]. Family members and close supporters of PWE also report high levels of stigma [57,60]. Increased levels of felt and enacted stigma are associated with lower levels of education, lower socioeconomic status, minorities, and those with less exposure to PWE [53,61]. Although low socioeconomic status is highly correlated with felt stigma, low socioeconomic status alone does not account for felt stigma. Leaffer, et al. [53] found that quality of life, depressive symptoms, and social support have the greatest impact on reported felt stigma in PWE. The researchers also identified that felt stigma is significantly associated with quality of life in low socioeconomic status individuals and with depression severity and social support in individuals with high socioeconomic status. Stigma, in all forms, limits the personal, educational, and social opportunities of the person with epilepsy, leading to a significant impact on the quality of life of both the person with epilepsy and his or her family members. 

The causes and consequences of epilepsy are heterogeneous across countries and regions, although there are significant knowledge gaps in many areas of the world including much of the Asian continent. It is clear that striking geographic differences exist in the etiologies of epilepsy [40,41,42]. There is as well cultural variability as to how a society relates to epilepsy [43,44,45,46]. The treatment gap found in low resource regions constrains the type and level of care that can be delivered to most of the people of the world with epilepsy since a majority of the worlds’ population lives in low resource countries. There are formidable political and economic forces responsible for the worldwide treatment gap in epilepsy, yet regardless of one’s ability to access appropriate treatment stigma continues to be an important factor in the life of PWE, and it is likely that stigma of epilepsy would need to be overcome in order to improve quality of life of individuals with epilepsy [9,19,47,48]. 

Projects in the developing world such as the Global Campaign against Epilepsy are currently working to change attitudes about epilepsy through education, to dispel myths, and reduce social isolation [62]. However, changes in attitude are very slow to develop. Evidence in the developed world points to seizure freedom as a critical factor to improving quality of life (QOL) in PWE, especially if achieved at an early age when social and cognitive skills are still developing [63,64]. The earlier in life seizures can be controlled the more likely it is that an individual will develop normal interpersonal skills and integrate into society (i.e., complete schooling, find work, and marry) [65]. Therefore, the most effective intervention to alleviate stigma is most likely to be elimination of the recurrent seizures.

### 1.5. Treatment of Medically Intractable Epilepsy

About 30% of PWE will fail medical treatment with ongoing and recurrent seizures despite medication, so-called intractable epilepsy. Intractable epilepsy has now been defined by the International League Against Epilepsy (ILAE) as recurring seizures that continue unabated despite trying and failing at least 2 anticonvulsant medications over at least one year [66]. Despite over 20 anticonvulsant medications available in North America and the European Union today for the treatment of epilepsy, the new medications are not more efficacious in controlling seizures compared to the old medications although newer medicines may have better side effect profiles. Intractable epilepsy, which is dangerous and life-threatening, significantly elevates mortality rates of PWE to 4.69 times that of age-matched controls [67]. The most effective means of reducing the morbidity and mortality of epilepsy is seizure control [68]. And intervention to treat medically intractable epilepsy with surgery to stop the seizures reduces mortality to that of the general population [67]. 

Surgery of epilepsy is a highly effective treatment to stop seizures for the majority of PWE [69,70]. The “low hanging fruit” of surgical treatment is surgery of temporal lobe epilepsy (TLE) due to the fact that TLE is common, frequently does not respond to medication, and has excellent opportunity to achieve seizure freedom from surgery. Boling et al. demonstrated in an epilepsy program established in Uganda, East Africa that surgery for medically intractable TLE can be accomplished in the developing world with good results on seizure freedom without serious complications [71]. In brief, the program recruited children with a history and seizure semiology typical for TLE from regional clinics of central and northern Uganda. Epilepsy characterization and seizure focus localization with video electroencephalography EEG, computed tomography CT brain imaging, and neuropsychology testing was done at CURE Childrens’ Hospital of Uganda (CCHU). The hurdles that must be overcome in any developing world approach to epilepsy treatment are related primarily to inadequate expertise for reliable EEG video interpretation, a shortage of available technology, and a harsh environment. The lack of expertise was overcome with remote analysis of video EEG and CT imaging in North America by epilepsy experts and teleconference linkage with the developing world site. The array of sophisticated technology available to epilepsy programs in the developed world will not be available in the foreseeable future to the majority of people in the world, who live in countries that are severely resource constrained. Therefore, only technology reasonably available in the developing world site can feasibly be used in the establishment of a sustainable epilepsy program. At the time the program was established in Uganda, there was no MRI available in the country but CT was available, which provided adequate imaging identification of mesial temporal sclerosis in 50% of the surgical patients. Likewise, the candidates selected for surgery were straightforward TLE and most likely to benefit from surgery, so-called low hanging fruit. The harsh environment relates to frequent power outages, temperature extremes, and lack of technicians to repair and upkeep sophisticated and sensitive equipment. The CT scan functions well in this environment and it was learned that video EEG does as well. The details of the methods and results of the CCHU epilepsy program are available at Boling, et al. [71]. This program established a paradigm for sustainable epilepsy care in a setting of severe resource constraints that optimized available technology rather than maximizing technological requirements. 

Fletcher, et al. then went on to analyze patients 8 years after an epilepsy evaluation at CCHU and found 70% were seizure-free after surgery, all the seizure-free patients had stopped their anti-epileptic medication, and none of the non-operated patients were seizure-free [72]. The authors retrospectively analyzed quality of life (QOL), stigma, and self-esteem of individuals both cured of epilepsy with surgery and non-operated with continued epilepsy. QOL was analyzed at long-term follow-up using an outcomes inventory developed and validated for people with epilepsy, the quality of life in epilepsy-31 inventory QOLIE-31 [73]. The test contains seven multi-item scales analyzing factors commonly impacting PWE and a single item that assesses overall health. The authors identified a significantly elevated QOL for patients in the surgical treatment group compared to non-surgical patients. Stigma was evaluated by Fletcher, et al. with a questionnaire developed by Joan Austin, et al. for children with epilepsy and their parents [74]. The Austin, et al. stigma measure has two scales, one for testing the child with epilepsy and another for the parent. The parent responds to five items on a 5-point Likert scale from 1 (strongly disagree) to 5 (strongly agree). To score, the five items are summed and divided by the number of items. A higher score reflects greater perceptions of stigma associated with their child having epilepsy. The child responds to 8 stigma related questions that ask how often they felt or acted in the ways described on a 5-point Likert scale from 1 (never) to 5 (very often). The questions broadly relate to subjective experiences with peer relationships, seizure and medication side effects, and day to day life challenges. To score, the items are summed and divided by the number of items. A higher score reflects greater perceptions of stigma. In this East African group of PWE, patients who were seizure-free after surgery showed significantly lower perceived stigma compared to non-surgical patients with continued epilepsy. The Austin parent survey likewise revealed lower perceived stigma in the parents with seizure-free children versus those parents with children who continued with seizures. 

Fletcher, et al. [72] found significant psychosocial improvement in individuals who realized seizure freedom after surgery for intractable epilepsy compared with those who continued with epilepsy, and similar psychosocial benefits were identified in the parents/caretakers of seizure-free individuals. The authors also identified that the developing world model of TLE surgery was similarly effective as the developed world experience to achieve seizure freedom, surgery had very low risks, and seizure-free results were robust at long-term follow-up.

## 2. Conclusions

Sub-Saharan Africa appears to have a very high overall prevalence of epilepsy. However, there is significant regional variation Asia and Africa in regards to epilepsy prevalence that may be related to the presence of endemic disease and parasites that are epilepsy etiologies and/or related to the availability of appropriate medical treatment of epilepsy. In the developed and developing regions of the world, stigma appears to be an important factor that affects QOL in PWE. In most of the developing world, there are differences between rural and urban populations in regards to degree of stigma experienced by PWE, and the degree of epilepsy-related stigma is largely reduced in a population by a higher level of education and acceptance of medical models of epilepsy etiology and treatment.

Medically intractable epilepsy is a chronic, disabling and dangerous disease. However, surgery will stop seizures in the majority of well-selected individuals, and seizure freedom will reduce the elevated mortality risk of epilepsy to that of age-matched controls [75]. Additionally, surgical treatment of drug-resistant epilepsy in the developed world improves QOL and reduces stigma [76,77]. These outcomes, which underpin an emphasis in the developed world to surgically treat intractable epilepsy when a clear opportunity for seizure freedom exists, are translatable to the developing and low resource regions of the world. Surgery is the only opportunity to cure intractable epilepsy, and, therefore, should be considered as an important treatment approach in high and low resource regions of the world to eliminate excess mortality of intractable epilepsy and mitigate the psychosocial consequences that impact individuals with medically intractable epilepsy.
